# Levels, trends and determinants of technical efficiency of general hospitals in Uganda: data envelopment analysis and Tobit regression analysis

**DOI:** 10.1186/s12913-020-05746-w

**Published:** 2020-10-06

**Authors:** Rogers Ayiko, Paschal N. Mujasi, Joyce Abaliwano, Dickson Turyareeba, Rogers Enyaku, Robert Anguyo, Walter Odoch, Pauline Bakibinga, Tom Aliti

**Affiliations:** 1 The Foundation for African Empowerment, P. O. Box 116, Arusha, Tanzania; 2 Department of Economics and Business, Universitat Pompeu Fabra, Barcelona School of Management, Balmes 132, 08001 Barcelona, Spain; 3 Makerere University Business School, Plot 21 A, Port Bell Rd, Kampala, Uganda; 4 Community Resource Development Initiative, P. O. Box 6653, Kampala, Uganda; 5 Department of International Public Health, Liverpool School of Tropical Medicine, Pembroke Place, Liverpool, L3 5QA UK; 6 Faculty of Health Sciences, Nile University, P.O Box 1070, Arua, Uganda; 7 East Central and Southern Africa Health Community, 157 Olorien, Njiro Road ECSA-HC, P.O. Box 1009, Arusha, Tanzania; 8 African Population & Health Research Center, Manga Close, Off Kirawa Road, P.O. Box 10787-00100, Nairobi, Kenya; 9 Ministry of Health, Plot 6, Lourdel Road, Nakasero, P.O Box 7272, Kampala, Uganda

**Keywords:** Data envelopment analysis, General hospital, Technical efficiency, Tobit regression analysis, Uganda

## Abstract

**Background:**

General hospitals provide a wide range of primary and secondary healthcare services. They accounted for 38% of government funding to health facilities, 8.8% of outpatient department visits and 28% of admissions in Uganda in the financial year 2016/17. We assessed the levels, trends and determinants of technical efficiency of general hospitals in Uganda from 2012/13 to 2016/17.

**Methods:**

We undertook input-oriented data envelopment analysis to estimate technical efficiency of 78 general hospitals using data abstracted from the Annual Health Sector Performance Reports for 2012/13, 2014/15 and 2016/17. Trends in technical efficiency was analysed using Excel while determinants of technical efficiency were analysed using Tobit Regression Model in STATA 15.1.

**Results:**

The average constant returns to scale, variable returns to scale and scale efficiency of general hospitals for 2016/17 were 49% (95% CI, 44–54%), 69% (95% CI, 65–74%) and 70% (95% CI, 65–75%) respectively. There was no statistically significant difference in the efficiency scores of public and private hospitals. Technical efficiency generally increased from 2012/13 to 2014/15, and dropped by 2016/17. Some hospitals were persistently efficient while others were inefficient over this period. Hospital size, geographical location, training status and average length of stay were statistically significant determinants of efficiency at 5% level of significance.

**Conclusion:**

The 69% average variable returns to scale technical efficiency indicates that the hospitals could generate the same volume of outputs using 31% (3439) less staff and 31% (3539) less beds. Benchmarking performance of the efficient hospitals would help to guide performance improvement in the inefficient ones. There is need to incorporate hospital size, geographical location, training status and average length of stay in the resource allocation formula and adopt annual hospital efficiency assessments.

## Background

Attainment of universal health coverage (UHC) – ensuring that everyone who needs health services gets them at sufficient quality without undue financial hardship – requires policies and programs that espouse effectiveness and efficiency in service delivery [1–3]. Efficiency is particularly important in low- and middle-income countries, which continue to grapple with high burden of common infections; maternal and child health complications; nutrition complications; epidemics and pandemics; and non – communicable diseases amidst resource constraints and rising healthcare costs [4–7].

Technical efficiency (TE) measures how well resources are transformed into outputs and how well health system goals such as improving health outcomes, responsiveness, fairness in financial contribution, quality and equity are achieved [8]. Efficiency is considered absolute when further optimization of any input or output of a decision-making unit (DMU) cannot be obtained without worsening any or some of its other inputs or outputs. On the other hand, efficiency is relative when measured among a set of comparable DMUs - a DMU with TE score of 100% is considered to be operating at the production efficiency frontier and thus efficient [6, 9, 10]. Scale efficiency (SE) if achieved when a DMU operates at its optimal size [11]. Changing the operational size of an efficient DMU either results in increasing returns to scale (IRS) whereby an increase in inputs results in a proportionally bigger increase in outputs (economies of scale) or a decreasing return to scale (DRS) in which increase in inputs creates a proportionally smaller volume of outputs (diseconomies of scale) [12]. Data envelopment analysis (DEA) and Stochastic frontier analysis (SFA) are the most commonly used approaches for conducting non-parametric and parametric TE analysis [13, 14].

Studies in Africa report varying levels of TE in health care facilities. Akazili et al. [15] found that over 60% of Ghanaian health centres were technically efficient while Zere et al. [16] found that the average TE score among hospitals in Namibia was 70%. An efficiency study of referral hospitals in Uganda estimates the average pure TE score of 91.4%, average SE score of 87.1% which translated to generating additional 45,943 outpatient visits and 31,425 inpatient days from the existing resources when efficiently used [17]. Various studies identify hospital size, ownership, bed occupancy rate, outpatient to inpatient ratio, average population income, technology, financing model, case mix and teaching status of hospitals as determinants of hospital efficiency [18–21].

The study setting was Uganda, a low-income East African country with a population of 40.3 million by mid-year 2019. About 21% of the population lives below the poverty line, physical access to healthcare within 5 km is at 86% and coverage by any form of health insurance scheme is less than 3%. In FY 2015/16, the government, external financing and out of pocket expenditure accounted for 15.6, 42 and 41% of the Total Health Expenditure (THE) per capita valued at US$ 51 [22–25]. The national development and health sector policy instruments – the second National Development Plan (2015/16–2019/20), second National Health Policy (2010) and the National Health Financing Strategy (2015/16–2024/25) – single out efficiency in healthcare delivery as a key determinant of progress towards national health and development goals [26–28].

The general hospitals that are the focus of this study are critical to the functioning of the health sector despite accounting for only 2.35% (163) of the 6929 health facilities in Uganda (see Table 1) [29]. In Uganda, they provide a wide range of preventive, promotive, outpatient, curative, maternity, inpatient healthcare services, general and emergency surgery, blood transfusion and laboratory services, training and research. General hospitals are the main primary healthcare (PHC) referral facilities linking the lower level health facilities to the regional and national referral hospitals and their service delivery standards put the number of beds and personnel at 100 to 250 and 185 respectively [30].

**Table 1:** Number of health facilities in Uganda disaggregated by level of care

Facility level	Count
Clinics	1578 (22.75%)
Level II Health Center (HCII)	3364 (48.49%)
Level III Health Center (HCIII)	1569 (22.62%)
Level IV Health Center (HCIV)	222 (3.2%)
General hospitals	163 (2.35%)
Regional referral hospitals	13 (0.19%)
Referral hospitals	3 (0.04%)
National referral hospitals	2 (0.03%)
Specialized hospitals	23 (0.33%)

Source: Ministry of Health. Facility Master List, 2018

About 64% of the government spending on health in the financial year (FY) 2015/16 was on health facilities (lower level facilities, general hospitals, regional referral hospitals, national referral hospitals and specialized institutions), and 38% of this expenditure went to general hospitals, 18% to private hospitals, 6.4% in regional and national referral hospitals and the rest to lower level health facilities [31]. Sixty-eight percent of general hospitals’ staff positions were filled while availability of key commodities stood at 83% [32]. Whereas higher-level referral hospitals registered improvements in the Standard Unit of Output (SUO) from 9,837,521 in 2015/16 to 9,956,067 in 2016/17, the overall SUO for general hospitals reduced from 17,692,056 in 2015/16 to 17,418,29 [32]. The SUO is a weighted average output of the most commonly performed procedures and services, considering their relative time and cost requirements [33]. A recent study of TE was carried out for referral hospitals in Uganda using the DEA approach, however, we could not find any for general hospitals yet they account for a huge part of the recurrent and capital expenditure [17, 31].

This study therefore, aimed at assessing the technical efficiency of general hospitals in Uganda. Specifically, the study; i) evaluated the levels of technical efficiency of general hospitals in Uganda in FY 2016/2017; ii) analysed the trends of technical efficiency of general hospitals in Uganda from FY 2012/2013 to FY 2016/2017; and iii) investigated the determinants of technical inefficiency of general hospitals in Uganda in FY 2016/17.

## Methods

### Study design

The study adopted a cross-sectional design to assess the technical efficiency of general hospitals in Uganda for FY 2016/17 using the input-oriented data envelopment analysis technique and a longitudinal design to analyse the trends in technical efficiency of general hospitals from FY 2012/13 to FY 2016/17.

### Study population

Seventy-eight (78) general hospitals (40 public and 38 Private Not for Profit [PNFP]) were selected out of the 114 general hospitals that routinely submitted health information through the District Health Information System-2 for FYs 2012/13, 2014/15 and 2016/17 because they either had complete data for the study variables or minor gaps in the data, which were ultimately filled in consultation with relevant authorities in the MoH, Uganda Catholic Medical Bureau (UCMB) and Ugandan Protestant Medical Bureau (UPMB). The sample size was considered adequate since it’s more than 3 times the combined number of inputs and outputs in the study and DEA primarily compares DMUs included in the study [34].

### Source of data and study variables

We abstracted data for the selected input, output and predictor variables from the Annual Health Sector Performance Reports (AHSPRs) for FYs 2016/17, 2014/15 and 2012/13 in an excel sheet [32, 35, 36]. Data on the number of staff was obtained from the National Integrated Human Resource Information System - iHRIS [37]. The variables selected for this study summarised in Table 2 below were informed by evidence from similar studies and availability of data; number of hospital beds and staff were considered as input variables while outpatient departments (OPD visits, admissions and health facility deliveries were selected as the output variables [15–17]. Predictor variables were environmental and institutional factors that have previously been shown to influence efficiency including hospital ownership, hospital size, number of staff, proportion of qualified staff, geographical location, bed occupancy rate (BOR), training status, outpatient visit ratio to total inpatient days, and average length of stay (ALoS) [18–21].

**Table 2:** Study variables and data sources

Variable	Definition	Measurement	Source (s) of data
**Inputs**			
Bed	Hospital beds	Total number of beds in the year	AHSPR for FYs 2012/13, 2014/15 and 2016/17
Staff	Medical personnel	Total number of staff (Medical Officers, Dental, Pharmacy, Nursing, Allied Health Professionals, Administrative and Other Staff) in the year	AHSPR (FY 2016/17), Integrated Human Resource Information System; Reports of the Catholic and Protestant Medical Bureaux
**Outputs**			
OPD	OPD visits	Total number of outpatient visits in the year	AHSPR for FYs 2012/13, 2014/15 and 2016/17
ADM	Hospital admissions	Total number of inpatient admissions	AHSPR for FYs 2012/13, 2014/15 and 2016/17
Deliveries	Deliveries (births)	Total number of deliveries in the year	AHSPR for FYs 2012/13, 2014/15 and 2016/17
**Predictors**			
Ownership	Hospital ownership	Authority that owns the hospital: public (1) or private (0)	AHSPR (FY 2016/17)
Hospsize	Hospital size	Size of the hospital classified using the median number of beds: large (> 120 beds [1]), small (<=120 beds [0]). Given the variability in sizes of general hospitals across the world, lack of global or national benchmark for their optimal size and the need to ensure fair distribution of small and large hospitals presentation in the 2 groups, the authors used the median bed size rounded to the nearest ten i.e. 120 as the benchmark to classify the 78 general hospitals as small and large.	PR (FY 2016/17)
Propqualstaff	Proportion of qualified staff	Number of staff with formal qualifications (Medical Officers, Dental, Pharmacy, Nursing, Allied Health Professionals, Administrative and Other Staff) as a proportion of all staff in the year	iHRIS, Reports from Catholic and Protestant Medical Bureaus
Region	Geographical location	Region where the hospital is located: Central or Western Uganda (1), Northern or Eastern Uganda (0)	AHSPR (FY 2016/17)
BOR	Bed occupancy rate	Total annual inpatient days as a ratio of annual available bed days × 100	AHSPR (FY 2016/17)
TrainingStatus	Training status	Hospital is used for training health professionals or not: Yes (1) and No (0)	Ministry of Health Training Unit, Catholic Medical and Protestant Medical Bureaus
OPDIBD	Outpatient visit to total inpatient days ratio	Total number of OPD visits divided by total number of inpatient bed days in the year	AHSPR (FY 2016/17)
AvStayADM	Average length of stay	Total annual number of inpatient days spent/total annual number of admissions	AHSPR (FY 2016/17)

### Model specification

The study is conceptualized based on the following health production function equation:

$$ \sum \limits_{i=1}^n{\displaystyle \begin{array}{c}\ \\ {} Qx1\dots Qxn\dots =\mathrm{f}\ \left(\mathrm{x}1,\mathrm{x}2,\mathrm{x}3,..\mathrm{xn}\right)\end{array}} $$

Where Q is the quantity of health output such as admissions, inpatient bed days, outpatient visits and deliveries while x_1_, x_2_ etc. are the production factors such as financing, personnel, and medicines.

The model specification followed the input-oriented DEA model based on both the Constant Returns to Scale (CRS) and Variable Returns to Scale (VRS) assumptions [8, 38, 39]. The CRS assumption of the Charnes, Cooper and Rhodes (CCR) Model facilitates calculation of the overall TE as a ratio of a reduced single ‘virtual’ value of output (s) to a reduced single ‘virtual’ value of inputs. On the other hand, VRS assumption of the Bankar, Charnes and Cooper Model (BCC) reflects the fact that general hospitals operate at different scales through introducing an additional.

constraint ${\sum}_{j=1}^n{\lambda}_j=1$ and variable, μ_O,_ into the CRS model thereby turning its straight-line efficiency frontier into a convex hull, which reflects variation in returns to scale. BCC model generates pure TE.

The CRS model assumed that each of the 78 hospitals produce the same, s number of outputs in various amounts, y_rj_ (*r* = 1, 2,. .., s = 3), using the same, m number of inputs in possibly different amounts, x_ij_ (I = 1, 2,. .., m = 2);

$$ \operatorname{minimize}\;\theta -\varepsilon \left(\sum \limits_{i=1}^m{s}_i^{-}+\sum \limits_{r=1}^s{s}_r^{+}\right), $$

$$ {\displaystyle \begin{array}{c}\theta {x}_{io}=\sum \limits_{j=1}^n{x}_{ij}{\lambda}_j+{s}_i^{-},\\ {}{y}_{ro}=\sum \limits_{j=1}^n{y}_{rj}{\lambda}_j-{s}_r^{+},\\ {}\mathrm{O}\le {\lambda}_j,{s}_r^{-},{s}_r^{+}\kern1em \forall i,j,r.\end{array}} $$

The VRS model also assumed that all the 78 DMUs produce the same, s number of outputs in various amounts, y_rj_ (*r* = 1, 2,. .., s = 3), using the same, m number of inputs in different amounts, x_ij_ (I = 1, 2,.., m = 2);

$$ \min\;{\theta}_o-\varepsilon \left(\sum \limits_{i=1}^m{s}_i^{-}+\sum \limits_{r=1}^s{s}_r^{+}\right), $$

subject to

$$ {\displaystyle \begin{array}{c}{\theta}_o{x}_{io}=\sum \limits_{j=1}^n{x}_{ij}{\lambda}_j+{s}_i^{-}\kern2em i=1,2,\dots, m,\\ {}{y}_{ro}=\sum \limits_{j=1}^n{y}_{rj}{\lambda}_j-{s}_r^{+}\kern2em r=1,2,\dots, s,\\ {}1=\sum \limits_{j=1}^n{\lambda}_j,\\ {}\mathrm{O}\le {\lambda}_j,{s}_r^{-},{s}_r^{+}\kern1em \forall i,r,j,\end{array}} $$

Whereby:

- y_rj_ is the output r produced by the jth DMU;
- x_ij_ is the input i used by the jth DMU;
- Ɵ_0_ is the efficiency score of hospital 0 under assessment; and
- ε > 0 is a non-Archimedean element smaller than any positive real number.

### Data management and analysis

A Microsoft Excel data base was constructed for all the relevant input, output and predictor variables using data abstracted from the Annual Health Sector Performance Reports of FYs 2012/13, 2014/15 and 2016/17 as well as the iHRIS. The datasets were validated by relevant authorities at the MoH and network of faith-based PNFP facilities. The excel data was exported to STATA version 15.1 to generate the descriptive statistics. It was then imported into the Efficiency Measurement System (EMS) [40] where the CRS and VRS TE scores were generated. SE was calculated as a ratio of CRS TE to VRS TE:

$$ \mathrm{SE}=\frac{constant\ returns\ to\ scale\ technical\ efficiency\ score}{variable\ returns\ to\  sca\mathrm{l}e\  technical\ efficiency\ score} $$

Trends in CRS TE, VRS TE and SE for the period FY 2012/13, 2014/15 and 2016/17 were analysed using excel in STATA. The VRS efficiency scores for FY 2016/17 were transformed into left censored inefficiency scores (ineffscores) using the formula below [41] and regressed against the following explanatory factors using a Tobit Regression Analysis: ownership, hospital size, proportion of qualified staff, geographical location, BOR, training status, OPDIBD and to ALOS:

$$ \mathrm{IneffScore}=\left(1/\mathrm{VRS}\ \mathrm{TE}\ \mathrm{Score}\right)-1 $$

The estimated Tobit model for the study was thus specified as indicated below;

$$ \boldsymbol{IneffScore}=\boldsymbol{\alpha} +{\boldsymbol{\beta}}_1\mathrm{ownership}+{\boldsymbol{\beta}}_2\mathrm{hospsize}+{\boldsymbol{\beta}}_3 proqualstaff+{\boldsymbol{\beta}}_4\mathrm{region}+{\boldsymbol{\beta}}_5\mathrm{BOR}+{\boldsymbol{\beta}}_6\mathrm{TrainingStatus}+{\boldsymbol{\beta}}_7\mathrm{OPDIBD}+{\boldsymbol{\beta}}_8\mathrm{AvStayADM}+\in \boldsymbol{i} $$

Where ***α*** is the intercept, ***β***_**1……**_ ***β***_**8**_ are the regression slope coefficients, and ∈*i* is a random error term. The objectives of regression analysis were to test the joint significance of all variables used and significance of the individual variables. The joint null hypothesis was tested using the likelihood ratio test and stated as H_O_: *β*_1_ = *β*_2_ = *β*_3_ = *β*_4_ = *β*_5_ = *β*_6_ = *β*_7_ = *β*_8_ = 0 while the alternative hypothesis was represented by H_A_: *β*_1_ = *β*_2_ = *β*_3_ = *β*_4_ = *β*_5_ = *β*_6_ = *β*_7_ = *β*_8_ ≠ 0. In testing for significance of individual variables (*β*_*n*_) using t- distribution test, the null and alternative hypotheses were represented as H_O_: *β*_*n*_ = 0 and H_A_: *β*_*n*_ ≠ 0 respectively.

## Results

### Descriptive statistics for the study variables

The study assessed technical efficiency of 78 general hospitals in Uganda. Descriptive statistics is provided for all inputs, outputs and predictor variables for FY 2016/17 as this is the focus year of comprehensive analysis. Descriptive statistics for input and output variables are provided in Table 3. The 78 general hospitals studied used a total of 11,092 beds and 11,416 staff to generate 3,020,147 outpatient visits, 697,946 admissions, and 165,932 deliveries in FY 2016/17. In the same period, the average number of beds and staff were 142 and 146 respectively while the average numbers for OPD visits, admissions, and deliveries were 38,720; 8948 and 2127 respectively**.**

**Table 3:** Descriptive statistics for input and output variables

Group	Variable	obs	Mean	Std. Dev	Min	Max
**A - All hospitals**	Beds	78	142	58.75402	61	305
	Staff	78	146	67.14357	42	433
	OPD	78	38,720	27,127.02	4873	178,146
	ADM	78	8948	4540.011	1427	23,560
	Deliveries	78	2127	1438.253	229	7002
**B – Public hospitals**	Beds	40	126	38.33315	76	224
	Staff	40	135	28.97035	81	204
	OPD	40	53,562	28,703.54	18,790	178,146
	ADM	40	10,972	3882.417	3885	23,560
	Deliveries	40	2776	1637.149	544	7002
**C – PNFP hospitals**	Beds	38	159	70.99094	61	305
	Staff	38	159	90.5376	42	433
	OPD	38	23,097	13,197.76	4873	64,580
	ADM	38	6817	4231.765	1427	20,446
	Deliveries	38	1444	738.1052	229	3453

There was wide variation in the annual volume of inputs and outputs across the country. On average, PNFP hospitals had 159 beds and 159 staff compared to 126 and 135 respectively in public hospitals. However, public hospitals had relatively higher volume of outputs with an average of 53,562 OPD visits; 10,972 admissions and 2776 deliveries compared to 23,097 OPD visits; 6817 admissions and 1444 deliveries respectively in PNFP hospitals.

Tables 4 and 5 respectively show the summary statistics of continuous and categorical determinants for technical efficiency of the 78 general hospitals in FY 2016/17. Overall, the average values for the proportion of qualified staff (formal qualifications), BOR, OPDIBD ratio and average length of stay were 76, 67%, 1.37 and 3.9 days respectively. The percentage of staff positions that are filled ranged from 43% in Matany hospital to 100% in Bwindi Community Hospital. The average BOR was 67% and ranged from 15% in St. Francis Hospital Nyenga to 178% in Apac hospital. OPDIBD ratio was highest in Busolwe (4.5) and lowest in Kagando (0.2). On average, patients were hospitalised for 3.9 days with the longest duration of 8 days at Matany hospital and the shortest duration of 2 days at Bududa, Busolwe, Comboni, Dabani, Kabarole, Kalisizo, Kamuli, Kyenjojo, Lyantonde, Naggalama and St. Francis Nyenga hospitals.

**Table 4:** Descriptive statistics for the continuous independent variables

Variable	obs	Mean	Std. Dev	Min	Max
**Propqualstaff**	78	76.05128	12.38652	43	100
**BOR**	78	66.61538	36.18503	15	178
**OPDIBD**	78	1.371795	0.9109575	0.2	4.5
**AvStayADM**	78	3.871795	1.399063	2	8

**Table 5:** Descriptive statistics for the categorical independent variables

Variable	Coding	Frequency	Percent	Cumulative %
**Ownership**	1, Public	40	51.28	51.28
	0, PNFP	38	48.72	100
**Hospital size**	1, Big (> 120 beds)	37	47.44	47.44
	0, Small (<= 120 beds)	41	52.56	100
**Geographical location**	1, Central or Western	42	53.85	53.85
	0, Northern or Eastern	36	46.15	100
**Training status**	1, Yes	28	35.90	35.90
	0, No	50	64.10	100

Frequencies of the categorical determinants summarized in Table 5 show that 40 of the 78 general hospitals are public-owned while 38 belong to the PNFP sub sector. Forty-one [41] hospitals had 120 beds or less while 37 had more than 120 beds. In terms of geographical location, 42 (53.85%) of the hospitals were either in central or western Uganda while 36 (46.15%) were either in the Northern or Eastern Uganda. Only 28 (36%) of the hospitals were recognized as training institutions for medical doctors, nurses, midwives, and allied health workers.

### TE of general hospitals in Uganda

Results of efficiency analysis for all the 78 hospitals during the FY 2016/17 summarized in Table 6 show that 97 and 90% of hospitals were inefficient under the CRS and VRS assumptions respectively while 97% were scale-inefficient. The average CRS, VRS and scale-efficiency scores were 49% (95% CI, 44–54%), 69% (95% CI, 65–74%) and 70% (95% CI, 65–75%) respectively. When analysed by type of ownership, the average CRS, VRS and scale-efficiency scores for public hospitals were 64% (95% CI, 59–72%), 82% (95% CI, 78–87%) and 78% (95% CI, 74–83%) respectively compared to PNFP hospitals whose scores were 73% (95% CI, 65–79%), 83% (95% CI, 77–89%) and 87% (95% CI, 81–93%) respectively.

**Table 6:** Hospital efficiency scores disaggregated by hospital ownership during FY 2016/17

Parameter in separate groups of hospitals	CRS TE 2016/17	VRS TE 2016/17	SE 2016/17
**All hospitals**			
Number of efficient hospitals	2	8	2
Number of inefficient Hospitals	76	70	76
Efficient hospitals (%)	3	11	3
Inefficient hospitals (%)	97	90	97
Average efficiency score (%)	49	69	70
Minimum score (%)	13	25	18
Maximum score (%)	100	100	100
**Public hospitals**			
Number of efficient hospitals	2	6	2
Number of inefficient hospitals	38	34	38
Efficient hospitals (%)	5	15	5
Inefficient hospitals (%)	95	85	95
Average efficiency score (%)	64	82	78
Minimum score (%)	28	50	45
Maximum score (%)	100	100	100
**PNFP hospitals**			
Number of efficient hospitals	10	16	10
Number of inefficient hospitals	28	22	28
Efficient hospitals (%)	26	42	26
Inefficient hospitals (%)	74	58	74
Average efficiency score (%)	73	83	87
Minimum score (%)	28	43	30
Maximum score (%)	100	100	100

Table 7 shows the top 10 scale efficient hospitals in Uganda with their respective super-efficiency scores during FY’s 2012/13, 2014/15 and 2016/17 and Table 8 shows the bottom 10 hospitals over the 5 years ranked on the basis of their CRS TE scores. Iganga, Mityana, Tororo and Ibanda were among the most efficient hospitals over the 5 – year period while Abim, Buluba, St. Anthony’s Tororo, Virika, Amai Community and Kisiizi hospitals were among the least efficient over the same period.

**Table 7:** Top 10 hospitals in FYs 2012/13, FY 2014/15 and FY 2016/17

FY 2012/13			FY 2014/15		FY 2016/17	
SN	Hospital	SE Score (Super-effCRS)	Hospital	SE Score (Super-effCRS)	Hospital	SE Score (Super-effCRS)
1	Iganga	1.0000 (2.0224)	Iganga	1.0000 (1.800)	Iganga	1.0000 (1.977)
2	Busolwe	1.0000 (1.4456)	Busolwe	1.0000 (1.373)	Tororo	1.0000 (1.112)
3	Bwera	1.0000 (1.2163)	Mityana	1.0000 (1.087)	Kalongo	0.9956
4	Mityana	1.0000 (1.0771)	Kagadi	1.0000 (1.027)	Kitgum	0.9946
5	Masafu	1.0000 (1.0768)	Pallisa	1.0000 (1.016)	Mityana	0.9818
6	Tororo	0.9910	Ibanda	0.9958	Angal St. Luke	0.9795
7	Kitagata	0.9906	Tororo	0.9926	Bududa	0.9759
8	Moyo	0.9896	Kitgum	0.9888	Atutur	0.9752
9	Ibanda	0.9884	Angal St. Luke	0.9880	Entebbe	0.9661
10	Entebbe	0.9817	Nebbi	0.9744	Ibanda	0.9584

**Table 8:** Bottom 10 hospitals in FYs 2012/13, FY 2014/15 and FY 2016/17

FY 2012/13			FY 2014/15		FY 2016/17	
SN	Hospital	CRS TE	Hospital	CRS TE	Hospital	CRS TE
1	Matany	0.19	Aber	0.43	St. Francis Nyenga	0.13
2	Maracha	0.21	Abim	0.24	Amai Community	0.16
3	St. Francis Nyenga	0.21	Amai Community	0.30	Kiwoko	0.17
4	Kisiizi	0.21	Amudat	0.45	Virika	0.17
5	Buluba - Leprosy	0.23	Anaka	0.46	Kisiizi	0.18
6	Rugarama	0.24	Angal St. Luke	0.54	Rushere Community	0.19
7	St. Anthony’s Tororo	0.24	Apac	0.64	Buluba - Leprosy	0.19
8	St. Joseph Kitovu	0.25	Atutur	0.96	Villa Maria	0.19
9	Abim	0.25	Bududa	0.62	Nkokonjeru	0.23
10	Virika	0.26	Bugiri	0.64	St. Anthony’s Tororo	0.23

### Trends in technical efficiency of general hospitals

**Fig. 1:**
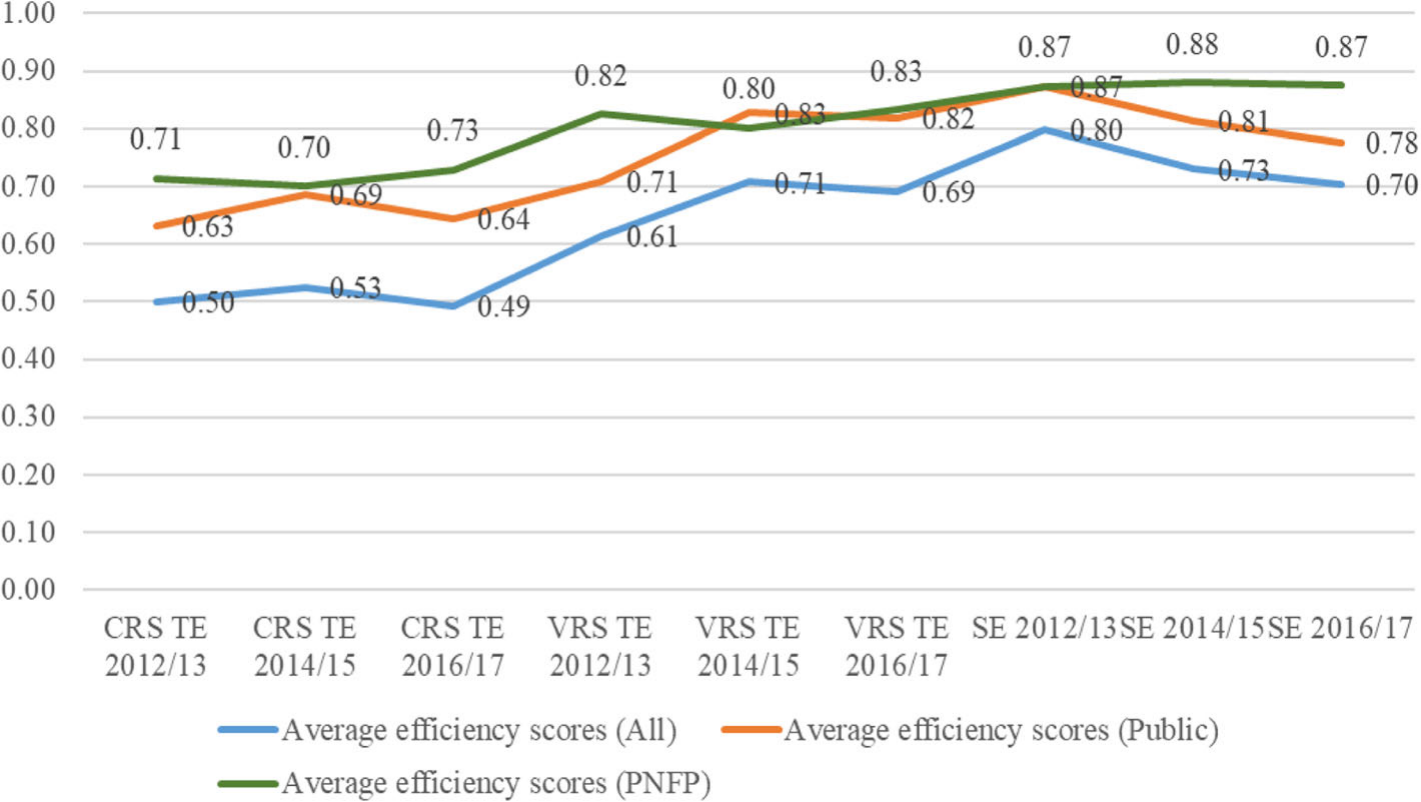
Trends in Average CRS, VRS and SE from FY 2012/13 to FY 2016/17

TE scores generally increased from FY2012/13 to 2014/15, and dropped during FY 2016/17. Figure 1 shows that the overall average CRS TE score of the general hospitals increased from 50% in FY 2012/13 to 53% in 2014/15 and reduced to 49% during FY 2016/17. The CRS TE score of public hospitals increased from 63% in 2012/13 to 69% in 2014/15 and reduced to 64% in 2016/17. However, PNFP hospitals registered a marginal reduction in CRS TE from 71% in 2012/13 to 70% in 2014/15 and an increase to 73% in 2016/17. Similar trends were recorded for the overall average VRS TE for the general hospitals increased from 61% in 2012/13 to 71% in 2014/15 and thereafter reducing marginally to 69% in 2016/17. The average VRS scores for public hospitals increased from 71% in 2012/13 to 83% in 2014/15 and then reduced to 82% in 2016/17 while the scores for PNFP hospitals reduced from 82% in 2012/13 to 80% in 2014/15 but rose to 83% in 2016/17. For all the general hospitals, the average SE reduced significantly from 80% in 2012/13 to 73% in 2014/15 and further dropped to 70% in 2016/17. SE remained fairly stable among PNFP hospitals but reduced among public hospitals.

### Determinants of technical efficiency of general hospitals in Uganda

The influence of the determinants of technical efficiency was analysed using the Tobit regression analysis technique: hospital ownership, hospital size, proportion of qualified staff, geographical location, bed occupancy rate, training status, outpatient visit ratio to total inpatient days and average length of stay.

Tobit Regression Analysis in Table 9 generates a chi square (X^2^) value of 51.19 for 9 degrees of freedom, which is significantly higher than the critical value of X^2^ for the same degrees of freedom i.e. X^2^ [9] = 16.919. Therefore, the joint null hypothesis that *β*_1_ = *β*_2_ = *β*_3_ = *β*_4_ = *β*_5_ = *β*_6_ = *β*_7_ = *β*_8_ = *β*_9_ = 0 is rejected at 0.05 level of significance (α), hence the alternative hypothesis (H_A_) that *β*_1_ = *β*_2_ = *β*_3_ = *β*_4_ = *β*_5_ = *β*_6_ = *β*_7_ = *β*_8_ = *β*_9_ ≠ 0 is upheld. An environmental factor with a positive co-efficient when the inefficiency score is regressed against it while holding the other factors constant shows that the level of inefficiency increases when the value of that factor increases.

**Table 9:** Output of Tobit Regression

VRSDEAIneffScore	Coef	Std. Error	t	***p*** > (t)	[95% Conf. Interval]	
					Lower	Upper
Ownership	−.2373533	.1566698	−1.51	0.134	−.5498213	.0751147
Hospsize	.3064594	.1306399	2.35	0.022**	.0459062	.5670126
Propqualstaff	−.0090919	.0050784	−1.79	0.078	−.0192205	.0010367
Geographical location	.2581932	.1243699	2.08	0.042**	.0101453	.5062411
BOR	−.0016612	.0021884	−0.76	0.450	−.0060258	.0027035
TrainingStatus	.2620071	.1297391	2.02	0.047**	.0032505	.5207637
OPDIBD	.0816522	.085905	0.95	0.345	−.0896799	.2529843
AvStayADM	.1948153	.0580116	3.36	0.001**	.0791149	.3105158
_cons	.2592612	.4617965	0.56	0.576	−.6617628	1.180285
Var (e. VRSDEAIneffScore	.2232797	.0383208			.1585589	.3144181

**Statistically significant at 5% level of significance

From the regression analysis, hospital size, geographical location, training status, and average days of inpatient stay were statistically significant determinants of hospital efficiency with positive coefficients. Inefficiency score was 0.3065 points (95% CI, 0.0459–0.5670) significantly higher in bigger hospitals compared to smaller ones while it was 0.2582 points (95% CI, 0.0101–0.5062) significantly higher among hospitals located in central or western Uganda compared to those in northern or eastern Uganda. Hospitals that trained personnel were less efficient than those that did not by 0.2620 points (95% CI, 0.0032–0.5208). A day’s increase in hospital stay increased inefficiency by 0.1948 points (95% CI, 0.0791–0.3105). OPDIBD had positive but insignificant co-efficient while hospital ownership, proportion of qualified staff, and BOR had negative co-efficient but none was a statically significant determinant of hospital inefficiency at α = 0.05. Even at a higher level of significance (10%), only the proportion of qualified staff was negatively correlated with inefficiency.

## Discussion

The 2010 World Health Report and several other reports estimate that between 20 and 40% of healthcare resources are wasted mainly through inefficient allocation and mix of resources, underutilization of inputs, corruption, uncontrolled overuse of certain services, and inefficient service delivery processes [2, 41, 42]. Assessing the efficiency of general hospitals which account for a huge part of the recurrent wage, recurrent non-wage and development resources in the order of 38% of the resources expended on health facilities in Uganda, is therefore critical for policy and programming [31].

The study assessed the levels, trends and determinants of TE of general hospitals in Uganda. Only 78 of the 114 general hospitals which had data on the study variables were included in the study. The overall average CRS, VRS and SE scores for the 78 hospitals were 49% (95% CI, 44–54%), 69% (95% CI, 65–74%) and 70% (95% CI, 65–75%) respectively. Only 2 of the 78 general hospitals were scale efficient meaning that 76 of them were not operating at the optimal scale. Separate analysis of efficiency for public hospitals returned CRS, VRS and SE scores of 64% (95% CI, 59–72%), 82% (95% CI, 78–87%) and 78% (95% CI, 74–83%) and those for PNFP hospitals were 73% (95% CI, 65–79%), 83% (95% CI, 77–89%) and 87% (95% CI, 81–93%) respectively, indicating some differences in efficiency between the 2 groups of general hospitals. The overlapping confidence intervals for each of the respective measures between public and PNFP hospitals indicates that there is no statistically significant difference in their levels of efficiency, similar to results from the tobit regression analysis.

Some hospitals such as Iganga, Mityana, Tororo and Ibanda were persistently among the most efficient while Abim, Buluba, St. Anthony’s Tororo, Virika, Amai Community and Kisiizi were among the least efficient over the analysis period of FY 2012/13–2016/17. The top performing hospitals have also been ranked similarly in past using the SUO, a technique commonly used by the Ministry of Health, Uganda [32]. Further analysis of how the most efficient hospitals mobilize, organize, deploy and manage resources and service delivery processes could guide implementation of relevant reforms across the country.

The lack of statistically significant difference in the efficiency of public and PNFP hospitals is not in agreement with majority of studies which show that public hospitals are more efficient than private ones because of having better input-mix, formal decision-making ability, regulated pricing of services and payment mechanisms [43–45]. The average VRS TE score of 69% means that on average, 31% of the general hospitals were VRS inefficient and could use 31% less resources to generate the same volume of outputs they are currently producing to remain relatively efficient. Therefore, 3,020,147 outpatient visits, 697,946 in patient visits, and 165,932 deliveries in FY 2016/17 could theoretically be produced even after reducing the number of beds and staff by 3439 (31% of the 11,092 beds) and 3539 (31% of 11,416 staff) respectively. The utility of the apparent “excess” beds and staff could be due to redundancies, absenteeism, presentism and or inefficient mix of resources. Given that most hospitals were operating under severe constraints of one or more of the inputs and some even work beyond their current capacity, it is advisable that attempts to optimize efficiency focus on improving performance at micro, meso and macro levels of the health sector.

Trends of TE generally increased between FY2012/13 and 2014/15, thereafter, dropped by 2016/17. CRS TE score for all the hospitals analysed increased from 50% in 2012/13 to 53% in 2014/15 and reduced to 49% by 2016/17; VRS TE for all hospitals increased significantly from 61% in 2012/13 to 71% in 2014/15 and reduced marginally to 69% in 2016/17. Whereas it’s difficult to pinpoint the exact factors leading to this trend within the scope of this analysis, it may be explained by the increased efforts to achieve Millennium Development Goal targets by the MDG and the “effort dilution” during the transition to the arising SDGs [46, 47].

The study found that increasing hospital size, location in central or western Uganda as opposed to northern or eastern regions; training health professionals and longer ALoS were positively and significantly associated with inefficiency. The finding in this study that larger hospitals were more inefficient than smaller ones was surprising since most studies including one on efficiency of Uganda’s referral hospitals, indicate that large hospitals were more efficient because they realize economies of scale through optimizing use of capital investments in infrastructure, technology and/or overheads [17, 48, 49], However, some studies show that smaller hospitals have a higher propensity of reaching the best practice frontier and thus are more efficient [50]. Whereas the actual size effect on efficiency may vary from one jurisdiction to another, economies of scale are generally expected in hospitals that have between 200 and 400 beds otherwise diseconomies crop in outside this range [51].

It is not surprising that hospitals located in Western or Central Uganda, which are more affluent parts of the country were relatively more inefficient than those located in Northern or Eastern Uganda – better socio-economic status is typically associated with higher effective demand and cost of care [20]. According to the Uganda National Household Survey 2017 [22], household poverty was highest in eastern region (42.7%) followed by northern (30.6%), central (22.7%) and then western region (19.1%). A study in Hong Kong found that public hospitals in richer districts had lower levels of efficiency that could be explained by people having better economic position and demand for high quality services which in turn drive up the volume and cost of inputs [52]. This finding is in agreement with another study in Canada which indicated that higher average population income was negatively associated with efficiency [19].

Regarding teaching status, the study finding is in tandem with several other studies which show that hospitals which act as teaching hospitals are more inefficient than their peers that only deliver health services [53]. Teaching hospitals provide more intense services, use higher volumes of resources, and adopt newer and more expensive technologies - characteristics explaining why they have higher proportion of complicated cases [54, 55]. In Uganda’s context where only 68% of general hospital staff positions are filled, dividing staff time for service delivery and teaching in addition to engaging students in service delivery is likely to increase inefficiency. However, it is not unusual to find comparable cost of care in teaching and non-teaching hospitals [56].

The ALoS being positive and significantly associated with inefficiency is not surprising since admissions are more resource-intensive and costlier than outpatient care, especially in terms of staff and bed requirements. Progressive reduction in ALoS in Apollo Gleneagles Hospitals increased productivity and savings of about US$ 0.9 million within 9 months [57]. A study of hospitalized children with Asthma, found that reducing ALoS from 2.9 to 2.3 days, reduced direct costs by $ 1543 per patient [58]. Another study in the United Kingdom found that reducing postnatal ward time by only 6 h (17%) could save about 8% of the costs [59]. However, the observed cost savings may simply mean that the costs have been transferred to families and communities besides reducing quality of care and increasing costs of treating complications [59, 60]. Therefore, efficiency interventions based on reducing ALoS must be carefully considered.

### Limitations of the study

The study has a number of limitations which might have affected the veracity of the findings. These include the exclusion of some variables such as quality of care and case mix due to lack of data, missing data and relatively weak quality of routine health information on which the national health sector performance reports are based. Some of the data limitations such as missing data or implausible data were addressed following consultations with relevant authorities in the Ministry of Health, UCMB and UPMB. This effort made it possible to maintain a relatively high number of general hospitals in the study out of the 114 that routinely submitted data on the study variables.

## Conclusion

The study provides empirical evidence on the level, trends and determinants of efficiency among 78 general hospitals that routinely submitted data of acceptable quality on the study variables. It shows that average CRS TE, VRS TE and SE scores among all the hospitals investigated were only 49, 69 and 70% respectively and that PNFP hospitals had higher technical efficiency scores than public hospitals, though the differences were not statistically significant. Furthermore, there was a general positive trend in efficiency over the period 2012/13 to 2016/17 under the CSR or VRS assumptions. Since the average VRS TE for all the hospitals was 69%, the volume of inputs needed to generate the current levels of outputs could theoretically be reduced by 31% i.e. 3439 less staff and 3539 less beds and redirected to other severely constrained facilities or functions in the sector.

The finding that some hospitals such as Iganga, Mityana, Tororo and Ibanda hospitals were persistently among the most efficient and other like Abim, Buluba, St. Anthony’s Tororo, Virika, Amai Community and Kisiizi were among the least efficient point to opportunities for targeted benchmarking and uplifting performance of poorly performing hospitals. Hospital size, geographical location, training status and ALoS that were found to influence efficiency should be of particular interest in policy making, planning and performance review processes in Uganda. The DEA is a good option for measuring hospital performance in addition to the SUO that has traditionally been used in the Uganda Annual Health Sector Performance Reports especially given its capacity to estimate the volume of inputs and outputs that can be optimized. We recommend to the Ministry of Health to include it in the tool kit for annual performance assessment, performance improvement planning and resource allocation.

## Data Availability

Data sets used in this study can be abstracted from the Annual Health Sector Performance Reports for FY 2012/13, 2014/15 and 2016/17 (https://www.health.go.ug/?s=Annual+Health+Sector+Performance+Report) and the Integrated Human Resource Information Systems (http://hris.health.go.ug/). Public access to the report and non-individualized part of the database is open. Administrative permission and formal ethical approval are only required if one seeks to analyse individual level data.

## Abbreviations

AHSPR: Annual Health Sector Performance Report
ALoS: Average Length of Stay
BOR: Bed Occupancy Rate
DEA: Data Envelopment Analysis
DMU: Decision Making Unit
TE: Technical Efficiency
CRS: Constant Returns to Scale
VRS: Variable Returns to Scale
SE: Scale Efficiency
PNFP: Private Not for Profit
SUO: Standard Unit of Output
OPD: Outpatient Department
IPD: Inpatient Department
